# Bioprinted Vascularized Mature Adipose Tissue with Collagen Microfibers for Soft Tissue Regeneration

**DOI:** 10.34133/2021/1412542

**Published:** 2021-03-13

**Authors:** Fiona Louis, Marie Piantino, Hao Liu, Dong-Hee Kang, Yoshihiro Sowa, Shiro Kitano, Michiya Matsusaki

**Affiliations:** ^1^ Joint Research Laboratory (TOPPAN) for Advanced Cell Regulatory Chemistry, Graduate School of Engineering, Osaka University, Japan; ^2^ Department of Applied Chemistry, Graduate School of Engineering, Osaka University, Japan; ^3^ Department of Plastic and Reconstructive Surgery, Graduate School of Medical Sciences, Kyoto Prefectural University of Medicine, Japan; ^4^ Toppan Printing Co., Ltd., TokyoJapan

## Abstract

The development of soft tissue regeneration has recently gained importance due to safety concerns about artificial breast implants. Current autologous fat graft implantations can result in up to 90% of volume loss in long-term outcomes due to their limited revascularization. Adipose tissue has a highly vascularized structure which enables its proper homeostasis as well as its endocrine function. Mature adipocytes surrounded by a dense vascular network are the specific features required for efficient regeneration of the adipose tissue to perform host anastomosis after its implantation. Recently, bioprinting has been introduced as a promising solution to recreate *in vitro* this architecture in large-scale tissues. However, the *in vitro* induction of both the angiogenesis and adipogenesis differentiations from stem cells yields limited maturation states for these two pathways. To overcome these issues, we report a novel method for obtaining a fully vascularized adipose tissue reconstruction using supporting bath bioprinting. For the first time, directly isolated mature adipocytes encapsulated in a bioink containing physiological collagen microfibers (CMF) were bioprinted in a gellan gum supporting bath. These multilayered bioprinted tissues retained high viability even after 7 days of culture. Moreover, the functionality was also confirmed by the maintenance of fatty acid uptake from mature adipocytes. Therefore, this method of constructing fully functional adipose tissue regeneration holds promise for future clinical applications.

## 1. Introduction

Soft tissue can be damaged by trauma, disease, high-grade burns, deep wounds, congenital deformities, or tumor resection. In such cases, large fatty tissue reconstruction should be readily available to provide an aesthetic structural and functional restoration, as well as for ameliorating patients’ psychological distress. These large defective adipose tissues, in particular following mastectomy, were traditionally replaced by artificial prostheses because of their easy and fast implantation, with a relatively short recovery time. Unfortunately, they have a limited lifespan of 10–20 years forcing patients to undergo additional surgeries. In addition, 40% of repeat surgeries occur within 5 years due to postimplantation complications such as infection, malposition, implant rupture, or foreign body immune response [1]. An additional safety concern has recently been raised about artificial implants because of their link with Breast Implant-Associated Anaplastic Large Cell Lymphoma (BIA-ALCL) occurrence [2], greatly reducing their clinical use.

Current approaches are therefore now focused on the use of autologous fat transplantation using the patient’s own adipose tissue from other body sites. However, these procedures involve several surgical interventions and are associated with a high risk of complications and donor site morbidity. They lead to large-volume resorption due to graft contracture or necrosis, sometimes up to 90% in long-term studies [3–7]. The major problem comes from insufficient graft revascularization after implantation, limiting oxygen and nutrient diffusion. Despite major advancements in microsurgery, notably by the development of the autologous tissue flap reconstruction which connects the graft microvasculature *in situ*, surgeons are still limited by the donor site availability and morbidity. Moreover, the surgical procedure is also costly and requires a high level of surgical skill, with failure still an occasional outcome (up to 2% rate) [8]. At this time, therefore, no suitable natural replacement is available to treat large adipose tissue defects.

It is here that 3D bioprinting appears as a promising solution, garnering immense interest over the past decade. Bioprinting enables the replacement of damaged tissues by facilitating the production of biosimilar constructs suitable for implantation, closing the gap between artificially engineered tissue constructs and native ones [9]. 3D printed tissues can be generated by codelivering cells and biomaterials with precise control of their composition and location. Therefore, while the large-volume fat graft implantations are limited by their nutrient and oxygen supply, depending on their diffusion via vascular structures, 3D bioprinted tissues could potentially overcome these numerous obstacles, providing a more *in vivo*-like structure with a natural degradation where shrinkage of the implant will be associated by its remodeling through the encapsulated autologous cells.

So far, few studies have been published to address the clinical need for a bioprinted restoration of functional adipose tissues. They generally used natural biomaterials rather than synthetic ones whose structure and composition are often associated with low biocompatibility or limited bioinductibility. Moreover, their additive content, such as unreacted monomers or catalysts, can induce immune body reactions, limiting their clinical uses. Of the natural bioprintable biomaterials, the best-performing ones directly use all the adipose extracellular matrix (ECM) components from the decellularization of native human adipose tissue in the bioink [10]. However, several disadvantages are associated with this approach including ethical limitations, donor site morbidity, and harsh treatment degrading essential ECM properties, as well as being an expensive and time-consuming method. Concerning the other natural biomaterials, alginate [11–14], gelatin [11, 13, 15–18], nanocellulose [12], and hyaluronic acid [16, 17] have already been used for bioprinting adipose tissues, but surprisingly, collagen type I which is the main constituent of native adipose tissue [19–21] was not included. All of these models relied on the adipogenesis of human adipose-derived stem cells (ADSC) after printing, ADSC being easy to isolate and use in tissue engineering. They have a major physiological limitation however, as only approximately 42% of ADSC can undergo adipogenic differentiation into the adipogenic lineage [22], in a time- and material-consuming process (usually requiring 2 to 3 weeks), without necessarily reaching a mature differentiated state. Differentiated ADSC still have a lower basal adipose metabolism compared to the direct use *in vitro* of mature adipocytes, whose identity and functions are comparable to native tissue [23, 24]. Therefore, an *in vitro* model of adipose tissue displaying fully mature functional adipocytes as in native adipose tissue is urgently needed.

For this purpose, using directly mature adipocytes has the benefit of improved efficiency and functionality with potentially less immunogenicity [25]. As they represent around 50% of the adipose tissue cell types [26], only a small piece of isolated tissue is needed to harvest large amounts of cells [25]. However, a major challenge exists for bioprinting mature adipocytes because of their fragile lipid unilocular morphology. The shear stress induced during the printing can negatively affect their function and viability during *in vitro* handling [27]. In a proof-of-concept study, Huber et al. tried to manually bioprint human mature adipocytes encapsulated in a photocurable methacrylated gelatin hydrogel [28], known for its controllable solution viscosity and cross-linking density. Although they showed viability maintenance of mature adipocytes for at least 5 days, this method was never confirmed with a proper bioprinter.

The next challenge is to overcome the current lack of a vascular network within the bioprinted adipose tissue which currently limits their transplantation as tissue transplant for patients. The aim is to get a final porous structure which allows cell infiltration and tissue ingrowth, while guaranteeing nutrient exchange by a promoted vascularization. Generally, oxygen, nutrients, metabolites, and catabolites have limited diffusion of less than a few millimeters through a scaffold [29]. Despite the general progress made in adipose tissue engineering, its vascularization still remains a critical issue, while the native adipogenic process is actually linked to an efficient vasculature in the tissue [30]. Generally, the addition of angiogenic growth factors and endothelial precursor cells is not compatible with adipocyte maintenance or even differentiation [31–33]. Recently, numerous techniques have emerged for the development of bioprinted vascularization in tissues [34, 35]. The techniques are often based on the direct microfabrication of channels and vessel-like structures, but it does not fit the complex vascular environment, lacking active vascular remodeling which is essential after implantation. Another way is to induce the self-cellular interactions to spontaneously generate capillary networks in the engineered tissues [36, 37], also called prevascularization. Upon implantation, anastomosis of these capillaries with the neighboring host vasculature occurs and ensures adequate blood perfusion to enable the graft survival. This method was thus used in this study, and the three cell types required to construct vascularized adipose tissue, namely, mature adipocytes, adipose-derived stem cells, and endothelial cells, were mixed in a collagen type I-based bioink. For more physiological maintenance of the mature adipocytes, collagen microfibers (CMF) were applied for their crucial biophysical and bioinductive characteristics [38], allowing the fragile mature adipocytes to be protected from mechanical shrinkage stress during the culture. Moreover, the CMF also provides a scaffold to induce the formation of the vascular network by endothelial cells [37]. Altogether, this method enables the fabrication of fully vascularized reconstructed adipose tissue showing host anastomosis following implantation for a higher graft survival rate [39].

Using our previous findings, the purpose of this study was thus to show for the first time the possibility of bioprinting viable and functional vascularized adipose tissues, including the fragile mature adipocytes, for a ready-to-use, high-benefit soft tissue regeneration. Overall, 3D bioprinters are available in three different forms: inkjet, extrusion-based, and laser-assisted [40], of varying costs and sizes. Some 3D bioprinters tend to be too large and expensive for widespread applications. An extrusion-based 3D bioprinter was used in this study, being generally of lower cost compared to the other bioprinter types. Other major limitations of traditional bioprinting approaches are that the fabrication of large tissues is generally not mechanically supported and that the ink tends to dry too quickly during long prints. To address these problems, embedded bioprinting using a supporting bath allows antigravity bioprinting of 3D constructs within yield stress and provides a humid environment which enables longer printing durations [9, 10, 41].

We firstly demonstrated the advantages of bioprinting using the gellan gum (GG) supporting bath for this purpose as compared to the classic patterning bioprinting on a surface. Then, higher size tissues showed the possibility of obtaining a fully vascularized structure throughout the bioprinted adipose tissue in an *in vivo*-like way, with a high cell survival where mature adipocytes were able to maintain their functionality for at least 7 days in *in vitro* culture following the bioprinting.

## 2. Materials and Methods

### 2.1. Materials

Porcine type I collagen was kindly donated from Nippon Ham (Osaka, Japan). Gellan gum (GG) (Mw = 500 kDa) was obtained from Sansho (Osaka, Japan). Fibrinogen (from bovine plasma, F8630), thrombin (from bovine plasma, T4648), bovine serum albumin (BSA, A3294), phosphate-buffered saline powder (PBS, D5652), collagenase from *Clostridium histolyticum* (type I, C0130), and Triton X-100 (T8787) were purchased from Sigma-Aldrich (St. Louis, MO, USA). Fetal bovine serum (35010CV) was purchased from Corning (Corning, NY, USA). Penicillin, streptomycin, BODIPY™ 500/510 C1, C12 (4,4-Difluoro-5-Methyl-4-Bora-3a,4a-Diaza-s-Indacene-3-Dodecanoic Acid (D3823)), goat anti-mouse secondary antibody Alexa Fluor® 647, and Hoechst 33324 (H3570) were obtained from Thermo Fisher Scientific (Waltham, MA, USA). The 4% paraformaldehyde (PFA, 16310245) and mouse anti-human CD31 antibody (M0823) came from Wako Pure Chemical Industries (Tokyo, Japan). The human umbilical vein endothelial cell (HUVEC, C25271) and endothelial growth medium (EGM-2MV, CC-3202) were purchased from Lonza (Basel, Switzerland). The Nile Red compound was purchased from Tokyo Chemical Industry (TCI, Tokyo, Japan). Dulbecco’s Modified Eagle’s Medium (DMEM) came from Nacalai Tesque Inc. (Kyoto, Japan). Cell-Based Propidium Iodide Solution (10011234) came from Cayman Chemical (Ann Arbor, MI, USA).

### 2.2. Collagen Microfiber Preparation

Based on our previous studies [36–38], the collagen microfibers (CMF) were made from the porcine collagen type I sponge, known for its good tolerance after implantation [42, 43]. It was first thermally cross-linked by dehydration condensation at 200°C for 24 h. Then, the cross-linked collagen sponge was mixed with ultrapure water at a concentration of 10 mg/mL (pH=7.4, 25°C) and homogenized for 6 min at 30,000 rpm (Violamo VH-10 S10N-10G homogenizer, diameter of 10 mm and length of 115 mm, AS ONE, Osaka, Japan). Then, the solution was ultrasonicated (Ultrasonic processor VC50, 50 W, 20 kHz, Sonics & Materials, Newtown, CT, USA) in an ice bath for 100 cycles (1 cycle comprised 20 sec ultrasonication and 10 sec cooling) and filtrated (40 *μ*m filter, microsyringe 25 mm filter holder, Merck, Darmstadt, Germany), before being freeze-dried for 48 h (Freeze dryer FDU-2200, Eyela, Tokyo, Japan). The obtained CMF was kept in a desiccator at room temperature.

### 2.3. Isolation of Mature Adipocytes and ADSC from Adipose Tissues

The 3 different patients’ human liposuctioned adipose tissues were isolated at Kyoto Prefectural University of Medicine Hospital. After a PBS wash, 8-10 g of tissue were minced to get fragments of around 1 mm^3^ in size using autoclaved scissors and tweeters, directly in the collagenase solution at 2 mg/mL in DMEM 0% FBS, 5% BSA, and 1% antibodies (sterilized by filtration). After one hour of incubation at 37°C at 250 rpm, DMEM was added and the lysate was filtrated using a sterilized 500 *μ*m iron mesh filter, before being centrifuged 3 minutes at 80g. The lysate was then washed two times in PBS with 5% BSA and 1% antibiotics and once in complete DMEM, by centrifugation between each wash. For the washing steps, the liquid fraction between the top layer (mature adipocytes) and the pellet (ADSC) was aspirated and discarded. Then, the pellet was resuspended in DMEM for ADSC expansion by changing the medium every two days and then by passaging the cells when they reach 80% of confluency. The mature adipocyte layer was moved to a new tube, and cells were counted in a 10 *μ*L isolated volume, by staining the nuclei for 15 minutes with Hoechst in DMEM and using a Turker Burk hematocytometer on a fluorescence microscope.

### 2.4. Fabrication of Bioprinted CMF Adipose Tissues

To construct the fat tissues, the CMF were first weighted and washed in DMEM without FBS before being centrifuged for 1 minute at 10,000 rpm to adjust the final concentration depending on the volume added to resuspend it (MiniSpin, Thermo Fisher Scientific, Waltham, MA, USA). When needed, the ADSC and the HUVEC were added after trypsin detachment (always used at passages 1-6) and centrifuged for 1 minute at 3500 rpm (MiniSpin, Thermo Fisher Scientific, Waltham, MA, USA) to get a final cell concentration of $2.5\times 10^{6}$ ADSC/mL and $1.25\times 10^{6}$ HUVEC/mL, as already reported in our previous study [39]. The pellet containing CMF, ADSC, and HUVEC was then mixed with the fibrinogen solution (to get a final concentration at 6 mg/mL, the stock solution was prepared in DMEM without FBS 1% antibiotics, to avoid the early gelation of the fibrinogen solution due to the FBS calcium ion content [44], then filtrated using a 0.2 *μ*m filter). Finally, the mature adipocytes were added at a final concentration of $3\times 10^{6}$ cells/mL and the tissues were bioprinted.

**Figure 1:**
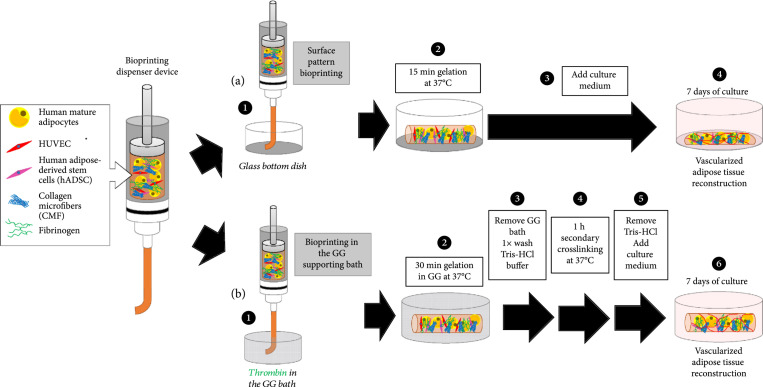
Patterning bioprinting on a surface and bioprinting in a supporting bath for 3D vascularized adipose tissue reconstruction. The same mixture containing the three types of cells (human mature adipocytes, HUVEC, and human adipose-derived stem cells) embedded in a fibrin gel including collagen microfibers (CMF) can be bioprinted using a mechanical extrusion dispenser bioprinter either as a pattern on the bottom surface of a dish (a) or inside a supporting bath made of gellan gum (GG) (b). After gelation, the two bioprinted tissues were cultured for 7 days to enable the reconstruction of *in vitro* vascularized adipose tissue.

For all the bioprinting steps (see Figure 1), syringes and nozzles used were sterilized with 70% ethanol and UV treatment. For the 2D patterning bioprinting on a surface, first, a mixture containing only the ADSC, the HUVEC, the fibrinogen, and the CMF in DMEM was prepared and thrombin was added at a final concentration of 3 U/mL (the stock solution was prepared in DMEM 10% FBS 1% antibiotics, filtrated using a 0.2 *μ*m filter) just before printing. The solution was then transferred to a glass syringe (1 mL Gastight Syringe Model 1001 TLL, 81320, Hamilton, Lancaster, PA, USA) with standard metal needles (Musashi Engineering, Inc., Tokyo, Japan). The syringe was set on a dispenser (NANO MASTER SMP-III, Musashi Engineering, Inc., Tokyo, Japan) using an adapter, and the dispensing system was kept at room temperature. The dispensing programs were designed on MuCADV (software for editing the dispensing pattern, Musashi Engineering, Inc., Tokyo, Japan) and completed debugging before dispensing. Two lines were printed on a 35 mm diameter glass-bottom dish, and the syringe and bed parts of the instrument were maintained at 4°C during the printing to defer the gelation of fibrinogen in the syringe. Then, a mixture containing the mature adipocytes, the fibrinogen, and the CMF in DMEM was prepared to bioprint an additional line between the 2 vascular lines. For the vascular and adipose lines, 20G and 15G needles were used to print the line structures, respectively. The printing parameters were at a dispensing speed of 1.28 mm/sec, with a moving speed at 10 mm/sec for the vascular lines and 2 mm/sec for the adipose line. The dish was then maintained at 37°C in the incubator to let the gelation occur for 15 minutes. Finally, the EGM-2 culture medium with 1 *μ*g/mL of insulin was added and renewed every 2-3 days, up to 7 days.

For the supporting bath bioprinting, first, gellan gum (GG) solution was produced by dissolving a 0.05-0.3 wt% gellan gum powder in PBS 1x solution and stirring at 85°C for 2 hours [45]. The solution is cooled to room temperature under slow shaking for future use. The supporting bath was then prepared by mixing GG with 10 U/mL thrombin before printing and was loaded into a 35 mm dish. Then, a mixture of the three types of cells was prepared, including the CMF and the fibrinogen in DMEM. Cell printing was then conducted inside the supporting bath maintaining the syringe and bed parts of the instrument at 4°C. The printing parameters were at a dispensing speed of 0.16 mm/sec, with a moving speed of 2 mm/sec and using a needle size of 16G. The dish was then maintained at 37°C in the incubator for 30 minutes for the gelation. The printed structures inside the supporting baths were incubated inside a sterile cabinet at room temperature for 1 h to ensure gelation. After gelation, the GG was gently removed by pipetting and was immersed in the 50 mM Tris-HCl buffer (pH 7.4) at 37°C for 1 hour, containing 15 U/mL of thrombin for a secondary cross-linking of the printed tissues. The thrombin solution was removed, and three washes in the Tris-HCl buffer only to dissolve the remaining GG were performed before adding the culture medium (DMEM for the nonvascularized adipose tissues and EGM-2 culture medium with 1 *μ*g/mL of insulin for the vascularized adipose tissues) with the renewal of the medium every 2-3 days.

### 2.5. Rheological Measurement

Viscoelastic testing was performed using the oscillating rheometer RheoStress 6000 (Thermo Fisher Scientific, Waltham, MA, USA) with a 0.052 mm gap using a 35 mm, 1° cone plate geometry (C35/1 TiL) at 20°C. Bioinks were prepared immediately prior to the measurement of the viscosity, as mentioned above, without or with cells (containing the three types of cells: mature adipocytes, ADSC, and HUVEC) and with different CMF concentrations (0, 0.5, 1.2, and 3.6 wt%), but constant fibrinogen concentration at 6 mg/mL. They were loaded into the gap between the parallel upper and lower plates of the rheometer. These bioinks were also compared to the native fat tissue from which was extracted the mature adipocytes and the ADSC. GG supporting baths were prepared with various concentrations. The rheological behavior of GG solutions was studied using the same instrument as for bioink characterization. To determine the yield stress quantitatively, steady rate sweeps were conducted by varying the shear rate from 0.01 to 10 s^–1^ and the time of each step. The shear rate was changed at 10 s^–1^ for 5, 10, 20, or 30 sec followed by a shear step at 0.01 s^–1^ for 10, 60, or 120 sec.

### 2.6. Mechanical Test

The elastic modulus of the cross-linked vascularized adipose tissues was measured with the EZ test (EZ/CE 500N, SHIMADZU, Kyoto, Japan). After mixing the adipose cells, the CMF (1.2%), the fibrinogen (6 mg/mL), and the thrombin (3 U/mL) in DMEM, the gelation was performed for 15 min. The gelated tissues were then put in a 24-well Transwell plate (0.4 *μ*m polyester membrane, Costar 3470, Corning, Corning, NY, USA) at room temperature for measuring and compared to *in vivo* real human adipose tissues cut at the same scale. A spherical mold (5 mm in diameter) was used to measure the elastic modulus at a head moving speed of 1.0 mm/min. The compressive test protocol was employed increasing the engineering strain until the testing stress to 200 mN. The modulus is automatically calculated by the EZ test in the elastic range (1-2 mN).

### 2.7. Viability Assessment

The viability of cells was quantified using the Live/Dead® viability assay kit (Molecular Probes®, Thermo Fisher Scientific, Waltham, MA, USA). After one PBS wash, the tissues were stained with calcein and ethidium homodimer-1 for 45 min at 37°C in the dark and then imaged using epifluorescence Confocal Quantitative Image Cytometer CQ1. Z-stack with the same steps and using the maximum intensity projection was performed keeping the same exposition time and excitation power for each sample. ImageJ software was used for the analysis of the projections, calculating the percentage of each staining.

### 2.8. Immunofluorescence and Immunohistochemistry Imaging

Tissues were fixed with 4% paraformaldehyde solution in PBS overnight at 4°C. They were then permeabilized in 0.05% Triton X-100 in PBS for 15 minutes and incubated for one hour at room temperature in 1% BSA in PBS. The first antibody mouse anti-human CD31 was added in BSA 1% and incubated overnight at 4°C. Finally, samples were incubated with the secondary antibody goat anti-mouse Alexa Fluor® 647 at room temperature in the dark for 2 hours and nuclei were counterstained with Hoechst as well as the Nile Red™ compound for intracellular lipid accumulation. The samples were rinsed in PBS and observed using epifluorescence microscopes (Confocal Quantitative Image Cytometer CQ1, Yokogawa, Tokyo, Japan, and Confocal Fluoview FV3000, Olympus, Tokyo, Japan). The phase contrast images were taken using Olympus IX71 (Tokyo, Japan). Images of the 3D reconstructed capillary and from inside one lumen were made using surface reconstruction on 3D volume images with the Imaris software (version 9.2.1, Bitplane, Belfast, UK). For histology staining, 3D tissues of 60 *μ*L using the same mixture of CMF, fibrinogen, mature adipocytes, HUVEC, and ADSC were seeded in a 24-well Transwell plate (0.4 *μ*m polyester membrane, Costar 3470, Corning, Corning, NY, USA) and cultured for 7 days before being fixed, rinsed 3 times in PBS, and sent to the Applied Medical Research company (Osaka, Japan) for paraffin embedding and CD31 immunostaining. The images were captured using an FL EVOS Auto microscope (Thermo Fisher Scientific, Waltham, MA, USA).

### 2.9. Fatty Acid Uptake Monitoring

For the fatty acid uptake monitoring by the adipocytes, first, the tissues were starved for 6 hours in DMEM without glucose and FBS, containing only 1% of BSA. 4 *μ*M of the fluorescently labeled fatty acid analog (BODIPY™ 500/510 C1, C12) was then added to the culture medium for a duration of 60 minutes with 10 *μ*g/mL of insulin to induce the fatty acid uptake, following Rogal et al.’s method [46], counterstained by Hoechst and Propidium Iodide (PI) for the dead cells. Imaging was performed on the living tissues, using the Confocal Quantitative Image Cytometer CQ1 at 37°C, and Z-stack images with the same steps and using the maximum intensity projection were performed keeping the same exposition time and excitation power for each sample.

### 2.10. Ethics Statement

The adipose tissues were collected from Kyoto Prefectural University of Medicine Hospital (Kyoto, Japan) from liposuction isolation of different female donors at the ages of 38, 71, and 72 and with BMI at 24.01, 22.6, and 26, respectively. All use was approved by the Human Ethics Committee (Approval number: ERB-C-1317-1) of Kyoto Prefectural University of Medicine Institutional Review Board and conformed to the principles outlined in the Declaration of Helsinki.

### 2.11. Statistical Analysis

Statistical analyses were performed using ezANOVA software (version 0.98, University of South Carolina, Columbia, SC, USA) by Tukey’s multiple comparison test (two-way ANOVA). When no marks are shown on the graphs, it means that the differences are not significant.

## 3. Results and Discussion

### 3.1. Patterning Bioprinting on a Surface

**Figure 2:**
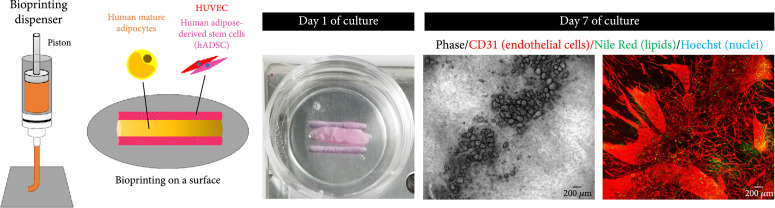
Patterning adipose tissue bioprinting on a surface. Patterning bioprinting was performed by printing two lines containing the vascular cell components (HUVEC and ADSC) surrounding a single line containing the mature adipocytes.

In our previous studies, we emphasized the importance of collagen microfibers (CMF) to provide stable and functional human engineered tissues [36–38]. These microfibers of around 20 *μ*m length, included in a fibrin gel, also allowed the bioprinting of vascular structures [37] from self-organized capillary network formation, displaying no tissue shrinkage. Our CMF-based bioprinting method, dispensing tissues on the surface of a culture dish, was therefore thought to be suitable for the production of an *in vitro* vascularized adipose tissue regeneration. First, similar line patterns, containing the cells required for the angiogenesis induction (ADSC and HUVEC) [39], were printed on a surface, incorporating a larger line in between containing the mature adipocytes in a fully cocultured scaffold (Figures 1(a) and 2). The tissue structure was maintained in the culture, even up to 7 days, but showed a settlement of the thickness, appearing like a 2D structure. Moreover, after immunostaining of the lipid content and the endothelial marker CD31, it appeared that despite the fact that angiogenesis was induced, some loss of mature adipocyte content was observed and they could not maintain a proper round shape, as seen in the phase image and in the limited Nile Red lipid staining. This could be due to their detachment from the printed tissue, which makes them float, due to their buoyancy property, or due to the fluidity of the bioink before gelation which makes the bioprinting of thick tissues difficult due to the gravity effect which induces its settlement [37].

Therefore, it was decided to proceed with the 3D bioprinting in suspension media, using a supporting bath, allowing the extrusion-based 3D printer device method to be used to its full potential. The bioprinting of tissue in a bath allows the possibility of printing non-self-supporting large-sized structures from low-viscosity bioinks into complex and well-defined structures, preventing their settlement and collapse [47]. This supporting bath printing technology has thus attracted interest over the past 5 years for complex tissue fabrication [48–50].

### 3.2. Properties of Gellan Gum Solutions as a Supporting Bath and CMF-Based Bioinks

For this purpose, the aim was first to use a suspension media which displays solid-like characteristics when no or low stress is applied and becomes liquid-like media when the bioprinting starts. Second, following the disturbance of the suspension media by the passing nozzle, the microstructure needs to spontaneously recover, permitting the transition from a fluidized state back to a solid-like state, thereby encapsulating the printed materials (cells and bioink). This property is called thixotropy. From this concept, we aimed to explore the potential of gellan gum as a support material for bath-enabled extrusion 3D printing of adipose tissues. Gellan is an extracellular microbial polysaccharide which can form tunable hydrogels, since its mechanical properties can be adjusted according to its concentration or by cross-linking multivalent cations, such as calcium ions [51]. Granular gellan gum (GG) hydrogels can form thixotropic gels and have thus recently been used for various bioprinting applications [51]. Compared to other supporting bath components, like gelatin or agar microgels, gellan rheology is generally stable over a range of temperatures. It is also a suitable support material for printing various types of bioink, while being easily removable by simple Tris-HCl washing dissolution [52].

Rheological measurements were performed to determine the most suitable GG concentration for the supporting bath during the bioprinting of 3D adipose tissues. Mature adipocytes are fairly deformable but require moderate shear stress to maintain their viability and functionality after bioprinting. As very few data are available concerning the possible acceptable range for the adipose cells, it was difficult to determine a suitable concentration for the GG bath solution.

**Figure 3:**
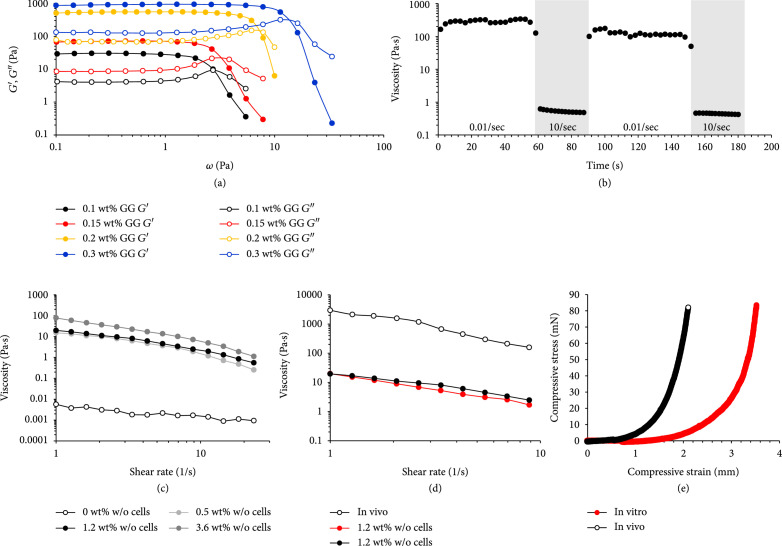
Rheological properties of different GG supporting bath concentrations and different CMF-based bioink concentrations. (a) Dynamic rheological characterization of the gellan gum supporting bath. Full and empty symbols represent storage moduli and loss moduli, respectively. (b) Thixotropic behavior of the 0.15 wt% gellan gum supporting bath following several cycles of high shear rates (10 s^-1^) and low shear rates (0.01 s^-1^). (c) Rheological viscosity properties of different CMF concentration bioink formulations. (d) Comparison of the bioink viscosities, containing or not containing cells and with the *in vivo* native adipose tissue. (e) Compressive stress measurements to determine Young’s modulus value, compared to *in vivo* adipose tissue.

From a cytometry study of mature adipocytes, Majka et al. [53] showed that it was possible to maintain their unilocular integrity when applying a typical flow pressure range of 5 to 10 psi (34-68 kPa), leading to a laminar flow within the cytometer of minimal shear stress for the cells, but no further study was performed to confirm this value as being suitable for mature adipocyte survival. Also, during the centrifugation steps following adipose tissue digestion, even though 80g speed (around 7.8 kPa) was applied, it permitted the maintained viability of the mature adipocytes. An overly high GG concentration in the supporting bath can result in a stiffer gel and correspondingly higher yield stress for the cells during the printing [51]. Since mature adipocytes are generally highly sensitive to mechanical stress [27], it was assumed that the GG bath concentration and bioink content should be chosen to ensure minimal shear stress for the cells. Different concentrations of GG bath solution (0.05-0.3 wt%) were thus prepared, and rheological analyses were performed (Figures 3(a) and 3(b)).

Storage ($G^{\prime }$) and loss ($G^{\prime \prime }$) moduli of the GG were measured in a shear stress sweep test (Figure 3(a)). Both $G^{\prime }$ and $G^{\prime \prime }$ increased along with the weight percentage of GG inside the supporting bath. The flow stress can be obtained from the crossover point of the $G^{\prime }$ and $G^{\prime \prime }$ curves. Once shear stress exceeded the linear region, the $G^{\prime }$ and $G^{\prime \prime }$ curves started to fall and meet each other at this flow point, where gel-to-sol transition occurs, necessary for the thixotropic property of the supporting bath for bioprinting purposes. This crossover point of $G^{\prime }$ and $G^{\prime \prime }$ shifted to higher shear stress upon increasing the concentration of gellan gum in the supporting bath. All GG preparations exhibited a shear-thinning behavior, as it was followed by a continuous drop with the increase in the shear rate. As shown in the graphs, the 0.1 wt% and 0.15 wt% concentrations of GG allowed a gel-to-sol transition for low shear stress Ѡ (below 10 Pa) and thus seemed more suitable for the embedded bioprinting of fragile mature adipocytes. The 0.2 and 0.3% concentrated GG thixotropic behavior required high shear stress that may damage the cells during printing or might even hinder a smooth printing process. Additional assays performed on these two concentrations highlighted the higher bioink diffusion through the GG bath at 0.1 wt% compared to 0.15 wt% (Supplementary Figure 1 and Video 1), leading to a less defined bioprinted construct. The latter concentration was thus chosen as the most suitable for the supporting bath bioprinting of *in vitro* adipose tissue.

The rheological characteristics of this 0.15 wt% concentrated GG supporting bath were further evaluated, as it influences the printing performance (Figure 3(b)). After the disturbance of a supporting bath microstructure by a passing nozzle and its displacement by the deposited bioink, the microstructure needs to spontaneously recover. This self-healing ability represents an essential feature of the matrix for it to be used as a supporting bath material. It permits the transition from a semiliquid state back to a solid-like state to enable the encapsulation of the deposited material. The thixotropic behavior of the 0.15 wt% GG supporting bath was monitored for 2 cycles. As seen in Figure 3(b), the viscosity of the bath decreased by applying high shear rate values (10 s^-1^), and the gel reassembly after strain release was instantaneous, recovering when the shear rate decreased (0.01 s^-1^). Moreover, this behavior did not change throughout the 2 cycles, and the GG bath was able to maintain its original viscosity after cycling. This property allowed us to print multilayered structures on the same path in the supporting bath. The 0.15 wt% GG bath was therefore deemed to be a suitable supporting bath for bioprinting adipose tissue.

Rheological measurements were then taken on the bioink formulations to characterize their rheological properties, as the viscosity will also influence their printability. The viscosity of different bioinks was investigated, using CMF at different weight percentages (0, 0.5, 1.2, and 3.6 wt%) in the same fibrinogen concentration (6 mg/mL), regarding the shear rate. Initial viscosity values increased along with CMF concentration (Figure 3(c)). All the bioinks containing CMF (0.5, 1.2, and 3.6 wt%) exhibited shear-thinning behavior, except for the bioink without CMF, which means that the CMF-based bioink should be suitable for the bioprinting approach. In addition, the increase of the CMF concentration was linked to an increase of the viscosity of the bioink, possibly exposing the cells to high shear stress which can alter their viability or function after printing [54]. Therefore, a moderate CMF concentration (1.2 wt%) was chosen. Interestingly, the addition of cells did not show a significant change of the viscosity values for the bioinks, as shown here for the 1.2 wt% CMF concentration, but they definitely showed lower values when compared to those of native fat tissue (Figure 3(d)). This confirms the unfeasibility of directly bioprinting the adipose tissue isolation, requiring its *in vitro* reconstruction in an optimized bioink.

**Figure 4:**
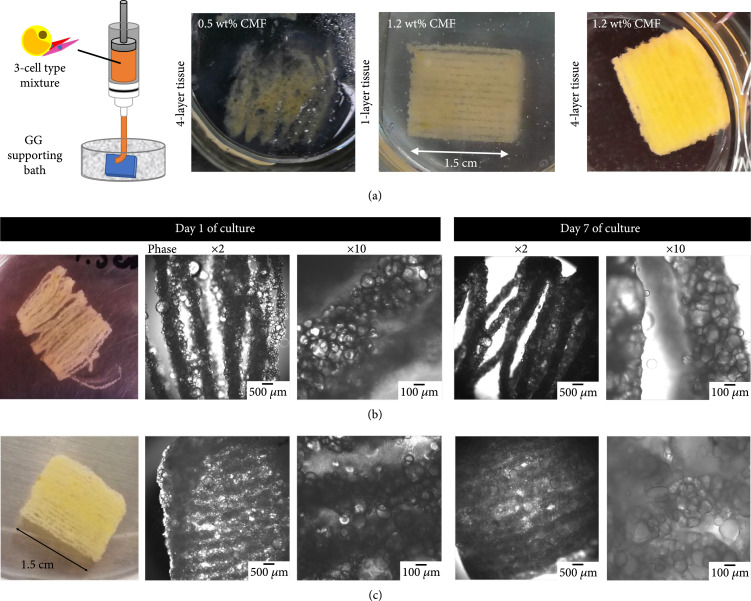
Square-shaped supporting bath bioprinting of adipose tissues with CMF-based bioink. (a) Representative pictures of the comparison between 0.5 and 1.2 wt% CMF concentrations in the bioink. The 1-layer tissue is a picture taken during the bioprinting. (b) Pictures and phase contrast images of square-shaped 4-layer adipose tissue with a 1 mm interline distance at several magnifications. (c) Pictures and phase contrast images of square-shaped 4-layer adipose tissue with a 0.8 mm interline distance at several magnifications. All these tissues contained the three types of adipose cells.

Finally, as the above rheological measurements on the bioinks were all performed without cross-linking of the tissue, which happens inside the GG bath during the bioprinting due to its thrombin content, thrombin was added to the mixture of the three types of cells encapsulated in 1.2% CMF with fibrinogen to measure the elasticity modulus just after full fibrinogen gelation. For the initiation of angiogenesis and the maintenance of the mature adipocytes in the scaffolds, adequate biochemical cues are also of importance to guide the cell-biomaterial interactions especially for adipose tissue, with suitable mechanical properties to mimic the mechanical response of native tissue. Young’s modulus of the *in vitro* tissues (Figure 3(e)) was 0.8±0.2 kPa, compared to the native *in vivo* adipose tissue at 2.9±0.5 kPa in accordance with the literature [55]. It should be noted that the *in vitro* tissues were not yet vascularized following the bioprinting, so the final elastic modulus should be enhanced, probably due to cell-induced gel shrinking during the vasculature formation (shrinking can be observed in Figure 4(b) between 1 and 7 days). This soft elasticity should provide structural integrity balanced by a possible scaffold degradation rate that supports adipose tissue regeneration after its implantation.

### 3.3. Supporting Bath Bioprinting Possibility of the Vascularized Adipose Tissue

The 0.15 wt% concentration was thus chosen for the bioprinting in the GG supporting bath. The two CMF concentrations of 0.5 and 1.2 wt% only were compared, the 0 wt% (only fibrin gel) being already reported in our previous study as not optimal for the vascular lumen formation with HUVEC cells, which is of importance for the implantation application to allow anastomosis occurrence [37] (video of the bioprinting in Supplementary Video 2). These two lowest concentrations of CMF are the ones which could be the most suitable for fragile mature adipocyte bioprinting, since the high-viscosity bioink is related to higher yield stress during the printing.

Figure 4(a) shows the result after 7 days of culture of 1.5 cm side size square-shaped bioprinted tissue with 1 or 4 layers. From these pictures, it appeared that 0.5 wt% CMF was not suitable for maintaining sufficient structural stability from the printing step (data not shown), and during the culture time, the tissue had almost completely disappeared, compared to the 1.2 wt% CMF which kept its square shape. The chosen distance between the printed lines of the total scaffold was also found to be of importance. The 1 mm distance (Figure 4(b)) resulted in a disrupted scaffold, where individual lines were displayed even if a high concentration of the mature adipocytes was still clearly observable within each line, while the 0.8 mm distance (Figure 4(c)) ensured the tissue shape stability from 1 to 7 days of culture, with the same high content of mature adipocytes inside.

### 3.4. Viability and Vascularization Assessment of the Bioprinted Adipose Tissues

**Figure 5:**
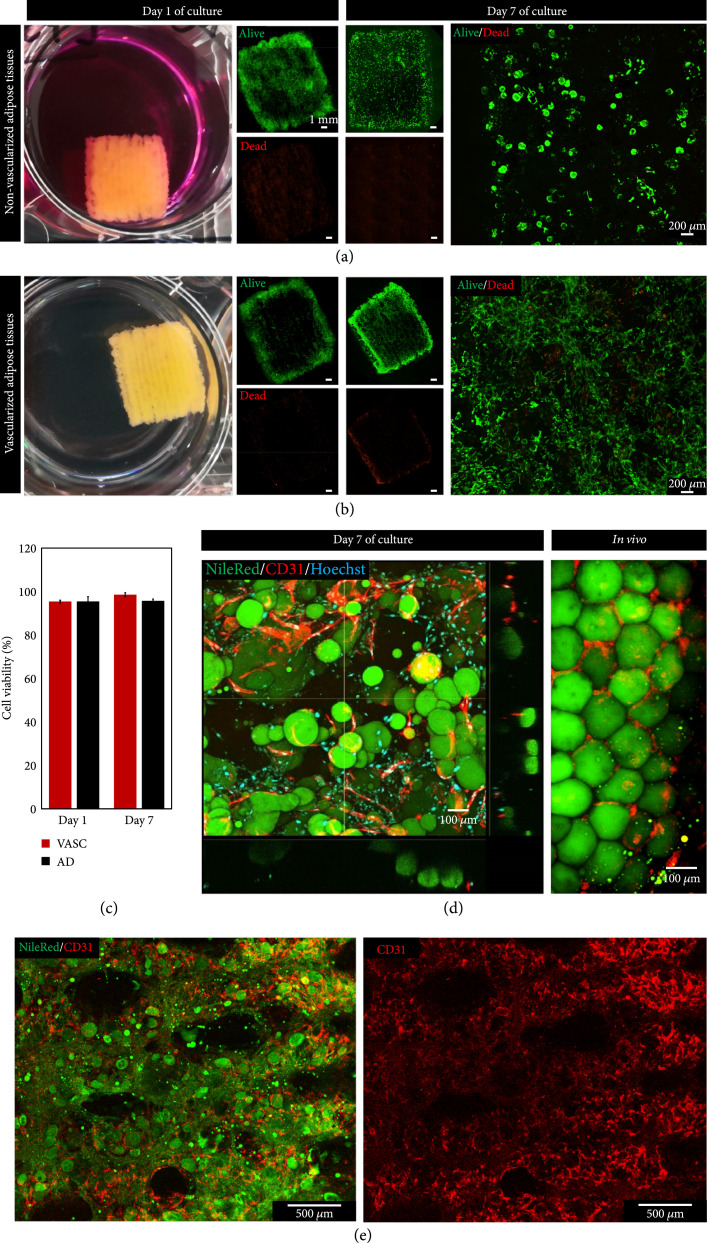
Viability and vascularization assessment of the bioprinted adipose tissues. (a) Representative images of the cell viability for bioprinted tissues containing only mature adipocytes. (b) Representative images of the cell viability for bioprinted tissues containing the three types of cells (mature adipocytes, HUVEC, and ADSC). (c) Quantitation of the cell viability from the Live/Dead images (AD: only mature adipocytes; VASC: the three types of cells). (d, e) Cross-section and representative images of fluorescent immunostainings of the CD31 endothelial cell marker, Nile Red lipid staining for mature adipocytes, and Hoechst for nuclei on bioprinted tissues and *in vivo*. These results come from three independent experiments with 1-3 tissues per experiment and condition.

**Figure 6:**
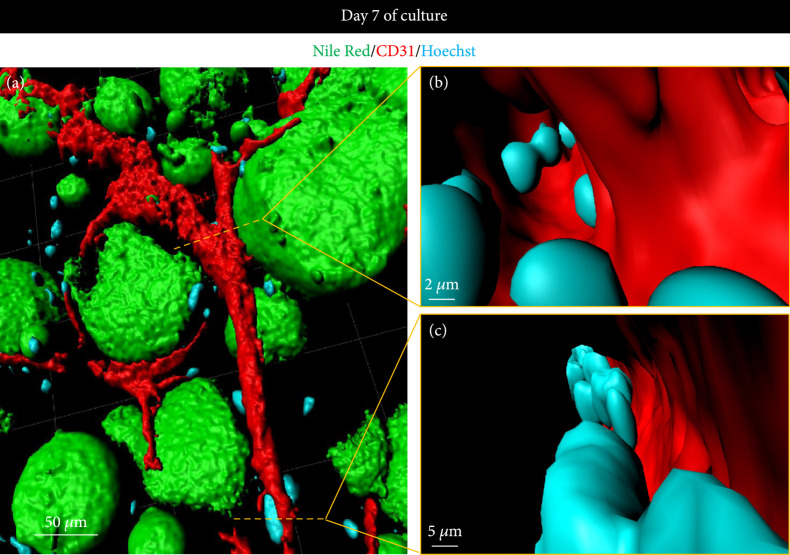
Lumen visualization in the bioprinted vascularized mature adipose tissues. (a) Representative Imaris software 3D surface reconstruction images from Nile Red/CD31/Hoechst immunostained bioprinted samples showing inside a blood vessel lumen at an intersection branching (b) and in the longitudinal visualization (c).

The next step was to confirm the cell viability in the final square-shaped bioprinted adipose tissue. Live/Dead assays were thus performed on days 1 and 7 of culture. Adipose tissues containing only mature adipocytes (Figure 5(a)) and adipose tissues also containing the two other cell types, HUVEC and ADSC (Figure 5(b)), were compared. It appeared that high viability was maintained for the two types of bioprinted adipose tissues for at least 7 days, with very few dead cells observed. The tissues containing only mature adipocytes displayed the typical round shape of the adipocytes throughout the tissues, while a large content of spindle-shaped living cells was observed in the tissues containing the three cell types, corresponding to the mature adipocytes, HUVEC, and ADSC. The fluorescence intensity quantitation of the Live/Dead assay (Figure 5(c)) confirmed the high cell survival with 95±2% and 95±1% of living cells on days 1 and 7, respectively, for the nonvascularized adipose tissues, and 95±1% and 98±1% of living cells on days 1 and 7, respectively, for the vascularized ones. The slight increase of viability in the vascularized adipose tissue could be due to the proliferation of the HUVEC and ADSC compared to the mature adipocyte tissues. This confirms that the angiogenesis that was induced to obtain vascularized adipose tissue, by endothelial growth factors added to the culture medium, did not alter the mature adipocyte viability. Immunostaining for endothelial marker CD31 and lipid vesicle fluorescent staining by Nile Red were then performed to highlight the full vascular network angiogenesis throughout the bioprinted tissue, which occurred during the 7 days of culture, providing vascularized adipose tissue similar to that *in vivo*, with capillary structures surrounding every single mature adipocyte (Figures 5(d) and 5(e)). The lumen structure of these blood capillaries was then confirmed using the 3D surface reconstruction of the bioprinted vascularized mature adipose tissue (Figure 6(a)) which allowed to navigate inside the vasculature lumen, with an example of a branching section (Figure 6(b)) and a longitudinal section where aligned nuclei can be observed (Figure 6(c)). Immunohistochemistry on manually seeded samples [39] (Supplementary Figure 2) also confirmed the lumen structures of different sizes in sectioned tissues, being observed in the vicinity (Supplementary Figure 2a) and surrounding (Supplementary Figure 2b) the mature adipocytes, structures which are expected to be the same in the bioprinted samples.

### 3.5. Functionality Assessment of the Bioprinted Adipose Tissues

**Figure 7:**
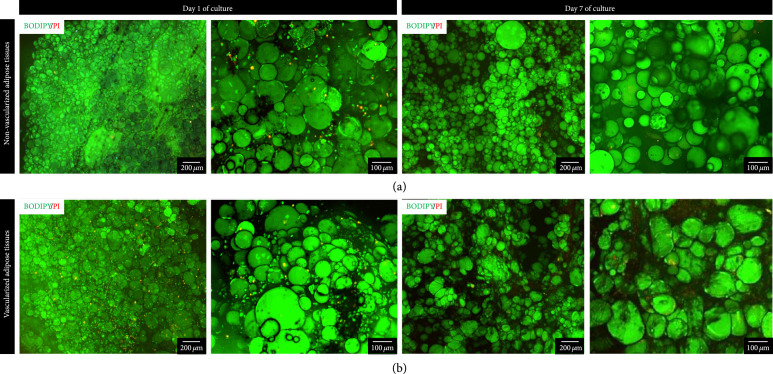
Mature adipocyte functionality assessment of the bioprinted adipose tissues. Monitoring of fatty acid uptake and accumulation was realized by the addition of a fluorescent fatty acid analog (4 *μ*M BODIPY™ 500/510 C1, C12) following one hour of incubation with insulin induction, counterstained by Propidium Iodide (PI) for staining the dead cells. (a) Representative images of fatty acid uptake in tissues with only mature adipocytes. (b) Representative images of fatty acid uptake in vascularized adipose tissues containing the three types of cells. n=3 samples per condition were performed.

Finally, not only the viability but also the maintenance of adipose tissue functionality after bioprinting are important. Therefore, the specific ability of mature adipocytes to take up fatty acids from the culture medium and to store them inside their unilocular lipid vesicles was monitored (Figure 7), as they can perform the same influx from diet-derived free fatty acids *in vivo*. Following starvation, a fluorescently tagged fatty acid analog (BODIPY™ 500/510 C1, C12) was added to the culture medium, with insulin to induce its uptake by stimulating the translocation of fatty acid transporter 1 from intracellular vesicles to the plasma membrane [56]. The fatty acid metabolism was monitored in real time on living cells, by fluorescence microscopy, allowing a noninvasive assessment of the cellular uptake of fatty acids and their accumulation by the mature adipocytes. After one hour of incubation at 37°C, the two different bioprinted models of adipose tissues both presented a high amount of fatty acid analogs in their unilocular lipid vesicles, confirming the maintenance of mature adipocyte function at least 7 days after bioprinting and *in vitro* culture. Propidium Iodide (PI) counterstaining again confirmed the low mortality rate inside the printed tissue. This further reinforces the fact that the angiogenesis induction in the vascularized bioprinted model did not alter the specific mature adipocyte function or affect the maintenance of their viability.

## 4. Conclusion

We conclude that our method using the CMF-based bioink to encapsulate mature adipocytes, ADSC, and HUVEC during their supporting bath bioprinting to generate multilayered constructs is a promising method for *in vitro* adipose tissue regeneration. For the first time, we demonstrated the successful fabrication of an *in vivo*-like adipose tissue using directly the high-potential mature adipocytes, with a simple and cost-effective printing process. The obtained bioprinted tissues reproduced the desired structure of native adipose tissue, displaying mature adipocytes surrounded by a dense vascularized blood network, which should allow each cell to obtain efficient diffusion of nutrients and oxygen in the grafted tissue after host anastomosis thanks to their lumen structures. Thus, due to this vasculature network, in addition to the inner porous structure created by the assembly of the collagen microfibers [38], this methodology should allow printing larger scale tissues. A further check concerns the maturation of the printed structure which is generally a challenge for all tissue engineering technology. The aim is to get a functional vascular tree structure which includes both the macroscale and microscale vessels. Following implantation, it can be expected that this vasculature structure will be remodeled during the anastomosis to ensure the proper blood diffusion through the whole tissue. Further studies are needed to evaluate this formation of bigger diameter blood vessels. To control even more the final vasculature structure design, while in this manuscript only the simple printed structure was performed, it can be possible to create more complex tissue designs with detailed inner organization. One of the possibilities would be to print the endothelial cells separately from the mature adipocytes and ADSC, to thus assign them to a defined location, closely surrounding the mature adipocytes, as in the *in vivo* structure (seen in Figure 5(d)).

The nondestructive printing method reported in this work maintains not only the viability but also the functionality of the encapsulated mature adipocytes. Adipose tissue is now widely recognized as an important organ for the whole body, as it participates in the regulation of a broad range of biological functions [57]. Thus, the preservation of its functionality is of importance for its homeostasis following implantation and improving the graft survival, which is highly desired for the regeneration of extensively damaged adipose tissue.

While fully customized tissues can be printed by this method, enabling the reconstruction of the personalized shapes for the patients, the final current limitation now relies on the printing working space availability. Usually, the bioprinters are limited in their vertical printing more than the horizontal space area. But recently, additional motion stages were developed, expanding the range of the motorized table for the movement in the z-axis. To our knowledge, the current biggest size bioprinted tissue in the supporting bath is already in the centimeter scale [58], so it should then be possible to easily reach the breast tissue scale which is around 10 cm in diameter for a hemisphere of 300 mL volume average [59], even if some adjustments will be needed to overcome the possible pressure differences between the top and the bottom of the bath container.

To further address the significance of this research in terms of medical matters, while this model allowed us to mimic human physiology in a customized shape depending on the patient’s needs, we can wonder about the implantation method for these high-scale bioprinted tissues. Regarding their elasticity, they should be flexible enough to be inserted even in a small incision, in a similar way to the current artificial silicone breast implants in which Young’s modulus elasticity is in a range of 14-1500 kPa, so even stiffer [60–63]. The final advantage is that it should be possible to get a full noninvasive process for the patient from the liposuction which involves a small incision up to the reconstructed graft implantation. An interesting last point could be the possibility to cryopreserve the bioprinted tissues for additional subsequent implantations in case of long-term adjustments of the final graft volume following its resorption [39].

## Data Availability

Data will be provided if requested.

## Supplementary Materials

Supplementary 1 Supplementary Figure 1: several concentrations of GG were prepared for checking their viscosity and the diffusion of the bioink following the bioprinting. The GG solutions (0.1-0.3 wt%) were assessed at room temperature. The 0.3 wt% GG solution did not show any fluidity compared to the 0.1 and 0.2 wt%. The solution was briefly shaken to generate bubbles and kept overnight. 0.05-0.15 wt% GG solutions were used as a supporting bath, in which PBS+blue dye (trypan blue) was injected with a pipette. All the images were taken after 10 min of injection to check the diffusion property.

Supplementary 2 Supplementary Figure 2: Lumen visualization in the vascularized mature adipose tissues. (a, b) Representative histology paraffin section of CD31 immunostaining images of a manually seeded vascularized mature adipose tissue showing the lumens inside the *in vitro* tissues in the vicinity (a) and surrounding (b) the adipocytes.

Supplementary 3 Supplementary Video 1: video of the 0.15 wt% GG solution fluidity.

Supplementary 4 Supplementary Video 2: real-time video of the bioprinting of square-shaped vascularized adipose tissues in a 0.15 wt% GG supporting bath containing 1.2 wt% CMF and the three cell types.
