# Supplementary figures and images for: Bioprinted Vascularized Mature Adipose Tissue with Collagen Microfibers for Soft Tissue Regeneration
Source: Cyborg Bionic Syst. 2021 Mar 13;2021:1412542. doi: 10.34133/2021/1412542 (PMC9494725; doi:10.34133/2021/1412542)

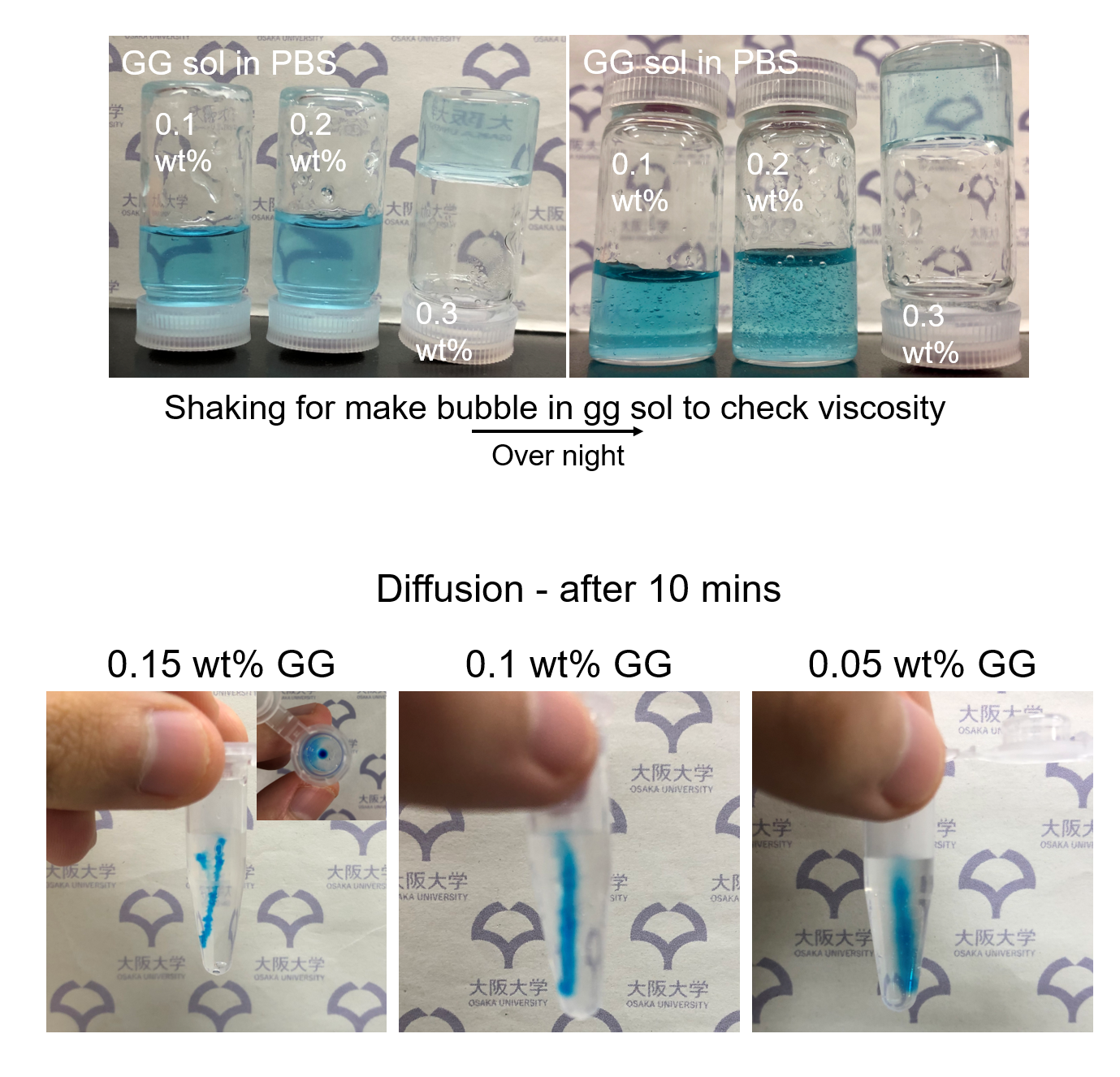

Supplement: Supplementary 1 — Supplementary Figure 1: several concentrations of GG were prepared for checking their viscosity and the diffusion of the bioink following the bioprinting. The GG solutions (0.1-0.3 wt%) were assessed at room temperature. The 0.3 wt% GG solution did not show any fluidity compared to the 0.1 and 0.2 wt%. The solution was briefly shaken to generate bubbles and kept overnight. 0.05-0.15 wt% GG solutions were used as a supporting bath, in which PBS+blue dye (trypan blue) was injected with a pipette. All the images were taken after 10 min of injection to check the diffusion property. [file 1412542.f1.tif]

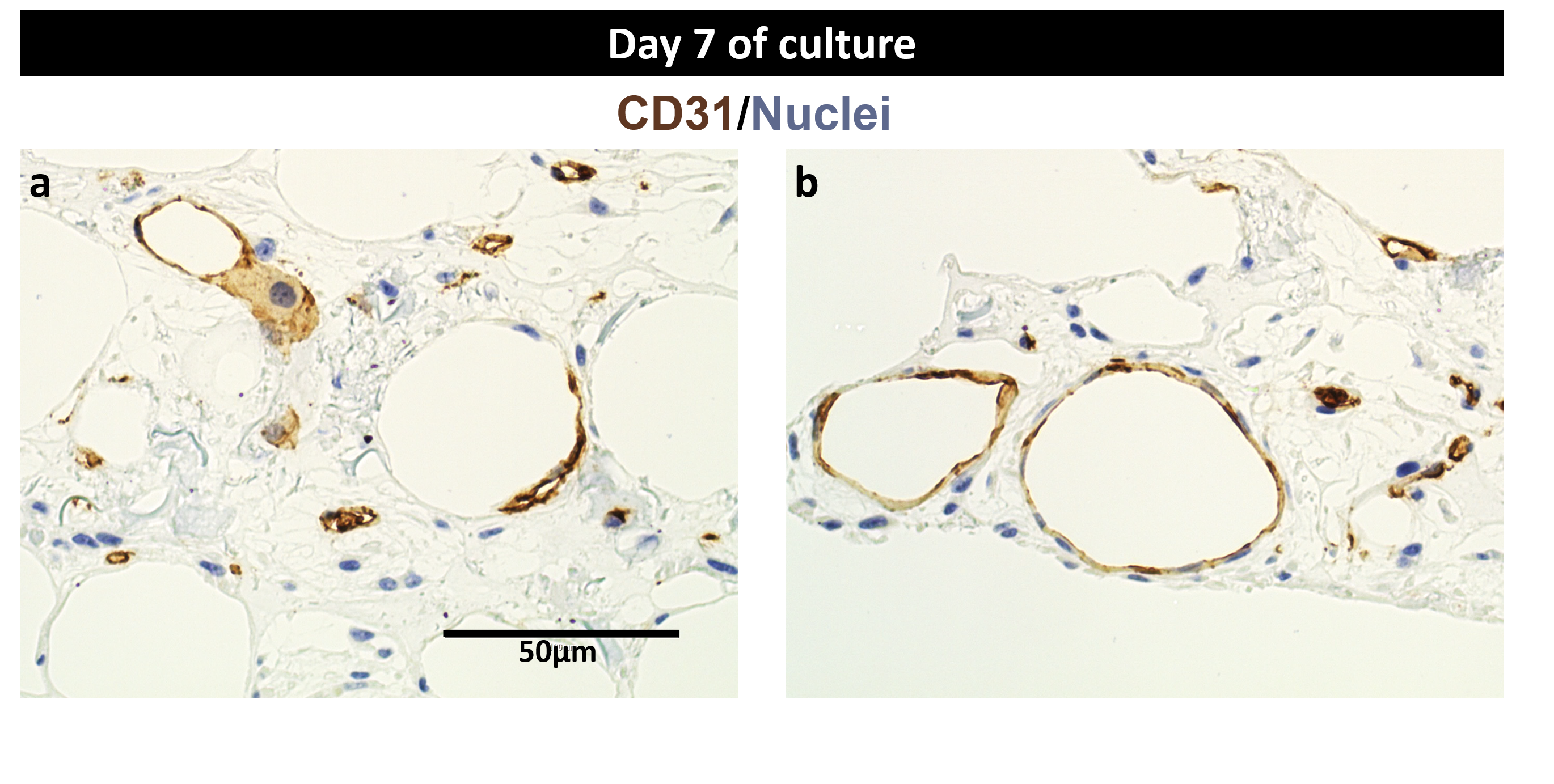

Supplement: Supplementary 2 — Supplementary Figure 2: Lumen visualization in the vascularized mature adipose tissues. (a, b) Representative histology paraffin section of CD31 immunostaining images of a manually seeded vascularized mature adipose tissue showing the lumens inside the in vitro tissues in the vicinity (a) and surrounding (b) the adipocytes. [file 1412542.f2.tif]
